# 2D Capsule Networks Detect Perceived Changes in Infant∼Environment Relationship Reflected in 3D Movement Dynamics

**DOI:** 10.21203/rs.3.rs-3088795/v1

**Published:** 2023-07-13

**Authors:** Massoud Khodadadzadeh, Aliza T. Sloan, Nancy Aaron Jones, Damien Coyle, J. A. Scott Kelso

**Affiliations:** 1 Intelligent Systems Research Centre, Ulster University, Derry/Londonderry, BT48 7JL, United Kingdom; 2 Human Brain and Behaviour Laboratory, Center for Complex Systems and Brain Sciences, Florida Atlantic University, Boca Raton, FL 33431, United States; 3 The Bath Institute for the Augmented Human, University of Bath, Bath, BA2 7AY, United Kingdom

## Abstract

Can infant exploration and causal discovery be detected using Artificial Intelligence (AI)? A recent experiment probed how purposeful action emerges in early life by manipulating infants’ functional connection to an object in the environment (*i.e*., tethering one foot to a colorful mobile). Vicon motion capture data from multiple infant joints were used here to create Histograms of Joint Displacements (HJDs) to generate pose-based descriptors for 3D infant spatial trajectories. Using HJDs as inputs, machine and deep learning systems were tasked with classifying the experimental state from which snippets of movement data were sampled. The architectures tested included k-Nearest Neighbour (kNN), Linear Discriminant Analysis (LDA), Fully connected network (FCNet), 1D-Convolutional Neural Network (1D-Conv), 1D-Capsule Network (1D-CapsNet), 2D-Conv and 2D-CapsNet. Sliding window scenarios were used for temporal analysis to search for topological changes in infant movement related to functional context. kNN and LDA achieved higher classification accuracy with single joint features, while deep learning approaches, particularly 2D-CapsNet, achieved higher accuracy on full-body features. For each AI architecture tested, measures of foot activity displayed the most distinct and coherent pattern alterations across different experimental stages (reflected in the highest classification accuracy rate), indicating that interaction with the world impacts the infant behaviour most at the site of organism∼world connection. Pairing theory-driven experimentation with AI tools thus opens a path to developing functionally-relevant assessments of infant behaviour that are likely to be useful in clinical settings.

## Introduction

Artificial neural networks (ANNs) were first developed to understand biological behaviour and mechanisms of cognition^1^. Turing believed that a crucial measure of artificial intelligence (AI) is the ability to mimic the way in which complex behaviour becomes organized in infants^2^. Given recent technical advancements in computing and AI as well as theoretical advancements in infant learning, it may be possible to use machine and deep learning techniques to study how infants transition from loosely structured exploratory movement to more organized, intentional action. Thus far, such methods have been restricted to the analysis of spontaneous movements^3^ and to distinguishing fidgety from non-fidgety movements^4^.

Though early infant movement is chaotic, meaningful patterns emerge as infants adapt to external perturbations and constraints through dynamic interaction between brain, body and environment^5,6^. However, little is known about the mechanisms by which infants begin to intentionally act on functional relationships with their environment. Laws governing bidirectional interaction between infant and environment are lacking, and the roots of conscious, coordinated, goal-directed action remain largely unexplored^6,7^.

A paradigm designed a half century ago to study infant memory and learning provides an experimental window into the formation of human *agency*, action towards an end^6^. In this so-called mobile conjugate reinforcement (MCR) paradigm, Rovee *et al*. connected a ribbon between an infant’s ankle and a mobile suspended over the infant’s crib^8,9^. Conjugate reinforcement refers to the sights, noises, and sensations due to mobile movement all being ostensibly dependent on and in proportion to the magnitude and rate of infant action. In short, the thinking was that the more the infant moved, the more ‘reward’ the mobile provided, stimulating further infant movement. Infants moved the connected leg at much higher rates compared to baseline, which Rovee and Rovee^8^ interpreted as reinforcement learning. However, mounting evidence suggests that rather than being rewarded by mobile stimulation *per se*, the increase in infant movement is driven by infant detection of the self∼mobile relationship^6,9–17^. The key variable manipulated in MCR is the infant’s functional connection to the world - transforming the infant from a disconnected observer to a connected actor. Bidirectional information exchange through coordination is thought to generate meaning and create the opportunity for infant discovery of agency^6,10,18,19^.

If infants do in fact discover that they can ‘make the world behave’ in MCR, dynamical analysis should expose the mechanisms of the discovery process. A necessary step in studying the development of conscious agency is being able to detect structural changes (in time, space and function) related to goal-directedness and to differentiate between exploratory and goal-directed action. One hypothesis is that the moment of agentive realization (‘Aha!) constitutes a kind of phase transition marked by sudden changes in activity rate, coordination and variability^10,18^. Although preliminary findings^20^ support the notion of agentive discovery as a phase transition, it may be possible for AI systems to automatically detect these and/or other changes in infant movement patterns reflecting detection of baby∼world causal relationships. Though the target measure in most infant contingency studies is movement rate, some studies have found that infants modify multiple features of movement including amplitude, timing^21^ and inter-joint coordination^22,23^ while exploring and exploiting their functional relationship with the mobile. AI tools may be particularly suited to deal with the complexity and subtleties of infant movement and, more generally, agent∼object interaction in 3D space.

A variety of machine and deep learning methods can be implemented for pose recognition using video recordings of infant movement. For example, McCay *et al*.^24^ extracted posed-based features from video recordings to develop an automatic and independent method to diagnose cerebral palsy (CP) in infants. They used a freely available library called OpenPose^25^ to obtain skeletal joint coordination during sequences of movement in 12 infants up to seven months of age^26^. That study achieved nearly 92% accuracy (two classes) using machine learning techniques (k-Nearest Neighbor (kNN) and Linear Discriminant Analysis (LDA)) and 91.7% accuracy using a fully connected convolutional neural network (FCNet). Additionally, Tsuji *et al*.^27^ constructed a neural network with a stochastic structure that was able to distinguish between normal and abnormal infant movements with up to 92.2% accuracy. (See also^3,4^).

In general, deep learning structures that use convolutional neural networks (CNNs) have effectively achieved state-of-the-art accuracy for classification of adult action^28–30^. In order to complete CNN feature extraction and classification automatically, regions of an output feature map are pooled. Though typically employed to reduce the computational cost of the model, such poolings may also result in a loss of important information. Two common approaches are max or mean pooling. In max pooling, a filter (a 2×2 grid, for example) is slid over the output feature map and only the maximum value in the grid area is retained. (In the example of a 2×2 filter, output data are reduced from four values to one). Although a useful means to simplify the network, it is impossible to know from poolings alone where and how many times a filtered feature is encountered in the data. A new form of neural network, the capsule network (CapsNet) was developed to address this issue^31,32^. Using groups of artificial neurons (*i.e*., mathematical functions designed to model biological neurons that encode visual entities and to recognize the relationships between these entities), CapsNets are designed to model part-whole hierarchical relationships explicitly. CapsNet encapsulates artificial neurons in its vector structure to arrange the first layer (primary capsules) and uses a novel Dynamic Routing (DR) procedure to create a perfect route between this layer and subsequent layers (parent capsules). In an image classification task, a hierarchical relationship is built into an object in the image so that it is possible to interpret features and determine which part of any image belongs to a particular object. For example, DR might allow a CapsNet to not only assess whether elements of a face (*e.g*., eyes, lips, nose) are present but also whether these elements are realistically situated in relation to one another. Lastly, CapsNets can be trained more efficiently than traditional CNNs. Because CNNs cannot handle rotational invariance, they must be trained on large amounts of input data which have been augmented by many combinations of transformations (*e.g*., rotation, zooming, cropping, inversion) to be able to classify new data accurately. Critically, since CapsNets can handle rotational invariance, they can be trained with fewer samples and may be particularly suited for infant action recognition for which large datasets are difficult to acquire^33^.

While techniques in motion identification, reconstruction, and analysis for automatic recognition of specific human activities continue to improve^34–36^, classifying human action patterns is challenging because these patterns often involve both temporal and spatial characteristics^37^. Joints connect different segments in the human body as an articulated system, and human actions comprise the continuous development of the spatial structure of these segments^34^. The majority of action recognition research focuses on adult movement, with just a small amount of research devoted to paediatric populations, including infants^38^. Researchers have begun developing systems to automatically analyze infant movement for early diagnosis of neurodevelopmental disorders^3,4,39^. However, automatic analysis of infant behaviour is complex since data are often captured in uncontrolled natural settings while the infant is freely moving and interacting with a variety of objects^40^. Handling information such as infants’ physical variations, lighting changes, and examiner involvement results in a lack of robustness in classification for markerless capture of movement data (*i.e*., estimating 3D limb position and movement from video recordings). Optical flow, frequency domain, and background removal are techniques commonly used to deal with these challenges^41–43^. While pose estimation from video recordings can deliver high accuracy, the extracted image sequences provide a large amount of data, but at a high computational cost.

Alternatively, reconstructed 3D skeleton data from marker-based motion capture (MoCap) systems have been shown to be dynamically robust and have anti-interference properties for automatic action classification^44^. Although consistent positioning of physical sensors (*i.e*., visual markers, accelerometers, gyroscopes) across infant participants is difficult, as joint landmarks are often obscured by fat, and the application of sensors may modify infant behaviour, skeletal joint information extracted from MoCap systems that use physical markers provides high temporal resolution and extremely high accuracy^45,46^. For example, Yu *et al*.^47^ used an adaptive skeleton definition to translate and rotate the virtual camera’s viewpoint to generate a new coordinate system. This produced a robust and adaptive neural network for automatic, optimal spatiotemporal representation. In many respects, action recognition is more straightforward using skeletal joint information than RGB video imagery, and hence is preferred here.

The data used in the present work were obtained from an MCR experiment^20,48^ which explored mechanisms underlying agentive discovery through analysis of infant motion, mobile motion and their coordination dynamics. Movement data were collected using a 3D MoCap system with motion markers placed on the infant’s body and on the mobile. Given the nature of this experiment and the fact that the MoCap data provide exact infant joint locations, several machine and deep learning approaches for classifying pose-based features are proposed and evaluated here. The first main objective is to classify movement across different experimental stages, ranging from spontaneous activity to reactions to a moving stimulus (here a mobile) to making the mobile move. Successful classification would indicate that the functional context and infant cognition drive changes in the structural features of infant movement. The second main aim is to study temporal features of infant behaviour using sliding windows. With this unique MCR dataset, we examined and optimized machine and deep learning methods to characterise cognitive processes and behavioural adaptation in infants. The classification accuracy of different experimental stages is intended to highlight the pros and cons of applying different machine and deep learning approaches in infant studies and, at the same time, expose whether behaviour is more constrained and characteristic within certain contexts, leading to higher accuracy classification. We demonstrate that approaches based on deep learning are well-suited for working with pose-based data. In particular, we show that CapsNet-based approaches, such as 1D-Capsule Network (1D-CapsNet) and 2D-CapsNet preserve the hierarchy of features and avoid information loss in the model’s architecture by substituting pooling with dynamic routing especially when fused features were employed. More to the point, we demonstrate that AI systems provide significant insight into the early ability of infants to actively detect and engage in a functional relationship with the environment.

## Methods

### Experiment

**Figure 1.**
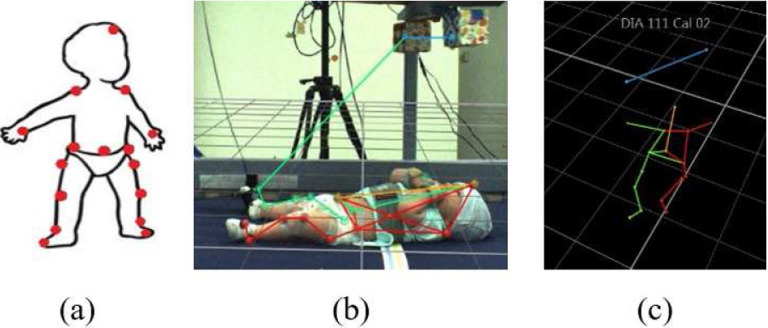
(a) Infant marker layout. (b) Infant in crib with mobile above. A 3D reconstruction of the markers is overlaid on top of the video recording. All coloured lines represent the virtual skeleton of the ‘baby-mobile object’ being tracked. The coloured dots are the reflective markers. (c) A 3D representation of another infant without the video feed.

Sixteen reflective markers were placed on 3–4-month-old babies (N=16) to track infant movement. All experimental protocols were approved by Florida Atlantic University (FAU) and Florida Department of Health (DOH) Internal Review Boards. This experiment was performed in accordance with FAU and Florida DOH guidelines and regulations. Informed consent was obtained from a parent and/or legal guardian of infant for both study participation and publication of identifying information/images in an online open-access publication. The marker arrangement can be seen in Figure 1(a). In each trial, the infant was placed in a crib face-up, with a mobile suspended above. The mobile consisted of two colourful blocks attached to the ends of a wooden arm. Reflective markers were affixed to opposite ends of the mobile. Seven infrared cameras surrounding the crib tracked each marker’s position at a rate of 100 Hz using a Vicon MoCap system. Three video cameras placed at different angles were also used to record each session. The experiment consisted of four experimental stages.

In the spontaneous baseline (2 min. long) the mobile was stationary. In the non-contingent baseline (2 min.), the experimenter triggered mobile movement independent of infant movement. Two strings were connected to the sock of one foot (the *trigger* foot, visible in Figure 1(b)), commencing the coupled or tethered phase (5–6 min.). The side of the body selected as the trigger foot was randomized. When the strings connected to the trigger foot were pulled, the mobile rotated. Infant trigger foot movement was digitally translated into rate of mobile rotation. The strings were then detached, leaving the mobile stationary once again constituting an untethered or decoupled phase (2 min.). These four stages respectively measure infant activity (1) when there was no mobile motion, (2) when mobile motion was simply a stimulus that was not affected by infant motion, (3) when mobile motion was a direct result of infant motion, and (4) when infant motion no longer resulted in mobile motion. An illustration of the extracted 3D infant skeleton is shown in 1(c).

### Pipeline overview

**Figure 2.**
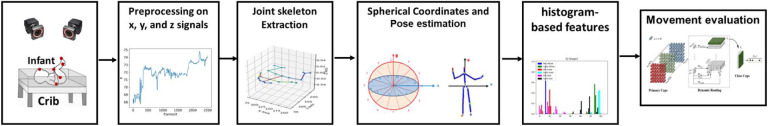
Overview of the feature extraction and classification steps.

An overview of the feature extraction and classification is shown in Figure 2. As will be described in the following section C, we first performed preprocessing to ensure the accuracy of the timeseries for the *keypoints*¸ the set of 3D movement markers extracted by the MoCap system. Important information was then extracted from preprocessed sequences categorized into distinct experimental stages (section D). In the next step, we used a spherical coordinate system to segment the 3D joints into an n-bin histogram (section E). A histogram-based approach was employed to create pose-based feature sets called Joint Displacements (JDs) to feed as inputs to the networks for movement evaluation (section F). The following sections of the paper explain each of these steps in detail. Finally, the machine (section G) and deep learning (section H) approaches tested here are described and justified.

### Dataset description and preprocessing

**Figure 3.**
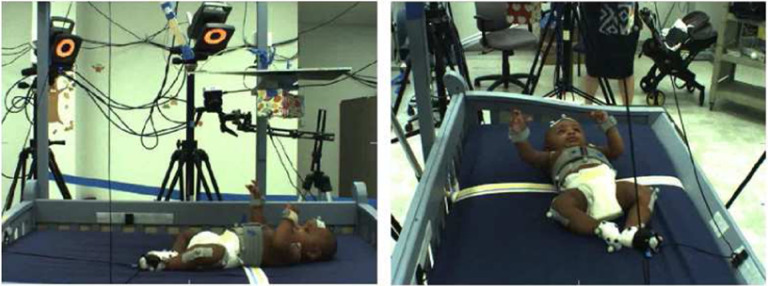
Different video camera angles for subject 1.

**Figure 4.**
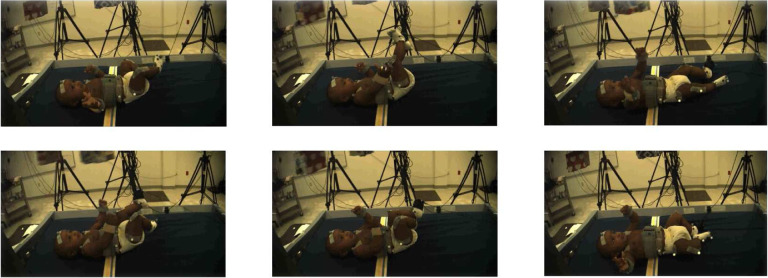
Different frames from one video camera viewpoint of subject 1.

**Figure 5.**
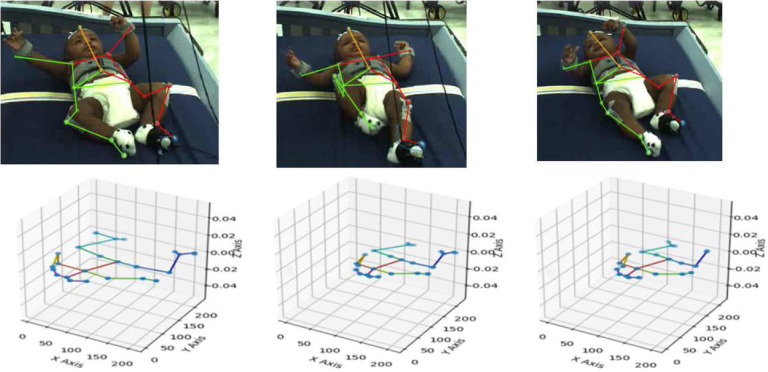
3D pose estimation using OpenPose for subject 1 for frames 1, 100, 150, respectively.

**Figure 6.**
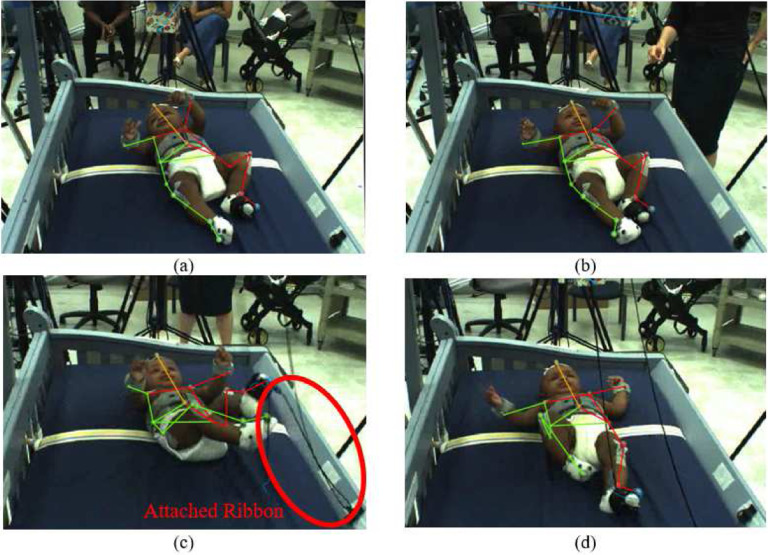
3D pose estimation during four different stages in the experiment: (a) Spontaneous baseline - no mobile movement (b) non-contingent baseline - experimenter moves mobile (c) Coupled/tethered stage (d) Decoupled/untethered stage.

The input data for baby movement pattern recognition are a sequence of 3D skeletal joint positions (*keypoints*) collected during the experiment using a marker-based MoCap system. For the current analysis, five infants were selected from the larger pool of participants because they had full datasets for all necessary markers throughout the experiment. The Vicon MoCap system used to collect these data provides a multi-view baby movement dataset as well as a three-dimensional skeleton nodes for baby movements that can compensate for RGB images’ disadvantages^49,50^. To visually introduce the data, Figure 3 shows one frame of different angles for subject 1. The position of the baby’s limbs changed constantly throughout the experiment (Figures 4–5). Figure 6 presents a single frame from each of the four stages. Figure 6 (a), (b), and (d) are examples where the ribbon is not connected to the trigger foot. In (a) and (d), the mobile does not move. In (b), the mobile moves when triggered by the experimenter. The baby can only activate the mobile once the ribbon has been attached to the foot visible in (c).

**Figure 7.**
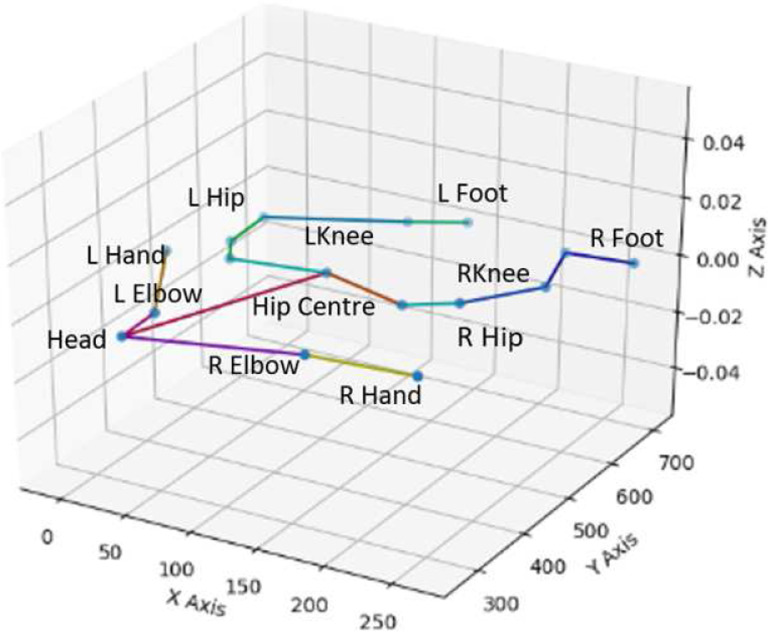
x, y, and z coordinates of body landmarks.

In every frame of data, the keypoints are labelled based on the anatomical locations of the movement markers in the frame. These keypoints are as follows: Head, C Pelvis, L/R Pelvis, L/R Shoulder, L/R Hand, L/R Hip, L/R Thigh, L/R Knee, L/R Ankle, L/R Foot (see Figure 7). However, a subset of these skeletal joints were included in the current study since complete data were available for only some of the markers. For the current project, we used marker data for 12 keypoints: Head, Center Pelvis, L/R Hip, L/R Shoulder, L/R Hand, L/R Knee, and L/R Foot. As a result, the dataset contains 12 3D sequences.

Various hurdles to 3D motion capture exist with infants such as marker jitters either due to system errors, extraneous reflections, or the mode of affixing markers to the infant’s body. For example, socks are made from stretchable fabric, allowing markers attached to the sock to wobble more than markers which are taped directly to the skin (a more rigid connection, that babies don’t particularly like). Other challenges include insufficient coverage of the infant’s limb motion either due to occlusion of the markers by vicissitudes of the experimental setup (*i.e*., at times, the mattress occludes almost 50% of the infant’s body, the mobile feedback system and its support beams) or by other body parts (*i.e*., stacking one foot on top of the other). The foregoing issues along with the complexity of the infant’s posture (*e.g*., crossing legs) can prevent the Vicon system from correctly identifying and tracking the keypoints.

Missing or incorrect marker data were handled using interpolation and filtering. Following that, we extracted features from the keypoint coordinate sequences that were preprocessed. Although keypoint missing or estimation errors may exist in a specific sequence input {*J*_*it*_(*x*,*y*) *i* = 1,2, …, 12}, there are a relatively small number of frames with errors in estimation. The three times standard deviation technique, where σ is the standard deviation of the sequence {*J*_*it*_}, was utilized for error elimination (1). Here *E*_*J*_ is the average of {*J*_*it*_}.


(1) $$J_{\text{it}}(x,y)=\{\begin{array}{l} J_{\text{it}}(x,y),\;\text{if}E_{J}-3\sigma \leq J_{it}\leq E_{J}+3\sigma \\ \text{nan},\;\text{if}J_{it}>E_{J}+3\sigma \text{or}J_{\text{it}}<E_{J}-3\sigma \end{array}$$

**Figure 8.**
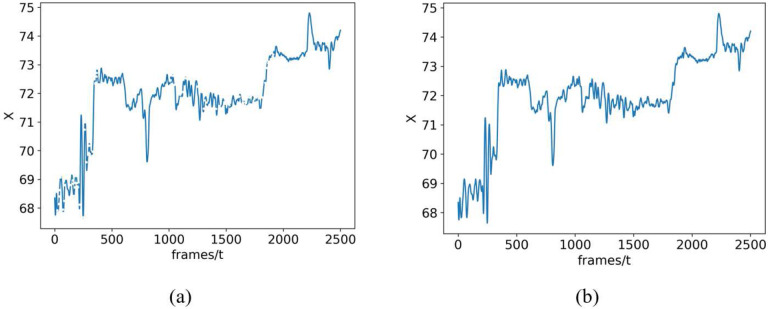
X coordinate plots of a single movement marker on infant’s right foot are examined both (a) before and (b) after preprocessing, showing that despite missing data points the overall integrity of the curves is preserved.

The trajectory of a joint’s location is continuous, and its velocity fluctuates uniformly during movement. Cubic spline interpolation was employed to fill in missing points. Ignoring frames with missing data may lead the actual movement to deviate from the subsequent calculation of movement complexity. Figure 8 depicts the infant’s right foot coordinate change curve during the time of around 2500 frames. Figure 8(a) illustrates the x-coordinate curve before interpolation with many discontinuous parts due to missing data. Figure 8(b) shows the same curve after interpolation indicating that the integrity of the data is preserved throughout this process.

### Dataset annotation

**Figure 9.**
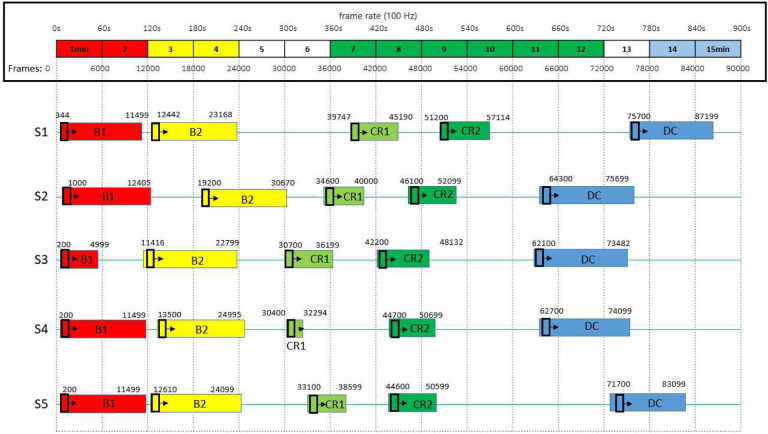
Experimental stages are depicted in different colours for five infants (S1-S5). In stage B1, the mobile is stationary. In stage B2, the experimenter initiates the movement of the mobile. In the coupled stages (CR1, CR2), the string is snapped to one sock and the mobile rotates when the string is tugged. In the final stage (DC), the string is detached, and the mobile is once again stationary. Red, yellow, light green, dark green, and blue represent stages B1, B2, CR1, CR2, and DC, respectively.

The video sequences were categorised as follows: B1 (spontaneous baseline, no mobile motion), B2 (non-contingent baseline, experimenter-triggered mobile motion), CR1 (the first minute of the coupled stage), CR2 (2 min. into the coupled stage - often the most active period in the coupled stage), DC (decoupled stage). The coupled phase was split across time (*i.e*., CR1, CR2) to investigate whether the various classification architectures employed here could differentiate between infant exploratory movement early in interaction from possible intentional activity later in the coupled phase. In each video, the five different experimental stages (B1, B2, CR1, CR2, DC) are labelled as 0 to 4, respectively. In subjects 1, 3, and 5, the ribbon was connected to the left foot; in S2 and S4, the right foot was connected. Figure 9 depicts the exact frame number at the start and finish for all five infants. Due to the nature of experiments with human babies, uneven stage lengths are inevitable (*e.g*., CR1 for S4 contains 1,894 frames rather than the planned 5,500).

### Spherical Coordinates of movement directions

**Figure 10.**
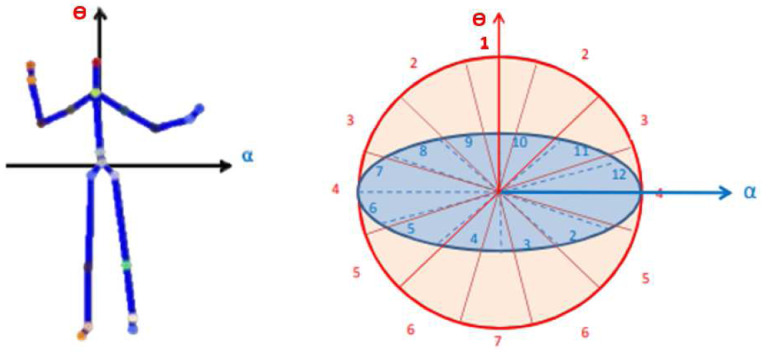
Keypoint locations are recalculated in reference to the pelvis to produce person-centred data (left). The partitioning of the 3D space into n bins is referenced by vectors alpha and theta (right).

Vicon data represent the position of markers relative to an arbitrary origin of the 3D capture space. A map of body-centric displacements can be created by designating a root joint marker and calculating displacement of other joints relative to the root. Here, we transferred the Center Pelvis to the centre of our spherical coordinates (the root) and then calculated all joint displacement between each joint and the centre (0,0,0). Thus, all analyses were run on joint displacements calculated from body-centered positional 3D motion capture data. A modified spherical coordinate system was then used to partition 3D space into *n* bins. Spherical coordinates align with the infants’ specific movement directions^1^. To elaborate, spherical coordinates are assigned to be view-invariant, meaning descriptors of the same type of infant position are similar even when collected from different perspectives (Figure 10). To create a compact representation of infant positions, we chose six informative joints (L/R hand, L/R knee, and L/R foot). The 3D space is partitioned into n bins by reference vectors alpha and theta, horizontal from L Pelvis to R Pelvis and perpendicular to the coordinate centre; therefore, any 3D joint can be localised at a specific bin^51,52^.

### Estimation of the poses using histogram-based features

Action recognition techniques based on histograms using a set of feature vectors extracted from the skeletal joint locations allow us to estimate 3D pose in lower dimensions relative to standard methods^25^ suited for pose estimation from 2D video data (*e.g*.,^53,54^). Histogram-based features derived from baby joint displacement have been successfully employed in a deep learning architecture to classify infant movements^55^. Using this approach, the displacement of each joint is extracted in a series of frames, and then a feature called Histogram of Joint Displacement (HJD) is obtained. HJDs track which particular joint occupies each spherical coordinate and for how much time. These HJDs are used as inputs for the machine/deep learning systems tested here.

### Classic Machine Learning approaches

We used k-Nearest Neighbour (kNN) and Linear Discriminant Analysis (LDA) as our classic machine learning approaches to classify the different stages based on changes in Histogram Joint Displacement. kNN uses the distance between each test point and *k* training points to determine which class best describes the data being tested. The test point is classified as belonging to whichever class of training data is most predominant in the *k* training points surrounding the test point. LDA models the decision boundary between classes. It identifies a linear combination of features that best differentiates the classes by maximizing the ratio of between-class variance to within-class variance. When applied to new data, this linear combination of features serves as a linear classifier. LDA operates on the Gaussian Distribution of input variables and can be used for both binary and multi-class classification. Both approaches have been previously applied to infant pose-based features^24^, providing a reasonable baseline for our evaluation performances.

### Deep Learning Approaches

Recent studies have demonstrated that deep learning frameworks can be successfully applied to human action recognition^30^. However, several obstacles exist, mainly the large amount of data required to enable deep learning and the difficulty of creating explainable AI when applying deep learning to infants’ activities and their healthcare domain^56,57^. Understanding how a framework makes decisions is critical in these domains. Yet, deep features in deep learning frameworks are often incomprehensible to humans. Hence, our deep learning frameworks employ hand-crafted pose-based feature sets to classify infant movements. The histogram vectors of the pose-based features were evaluated using different deep learning architectures, including a Fully connected network (FCNet), 1D-Convolutional Neural Network (1D-Conv), 1D-Capsule Network (1D-CapsNet), 2D-Conv, and 2D-CapsNet. A range of hyperparameters for each architecture was optimized using a cross-validation approach. The result section describes the model’s parameters and other statistics such as the size of the input and output layers, kernels, and the number of filters.

#### Fully connected networks

**Figure 11.**
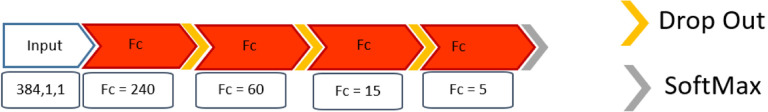
The proposed FCNet architecture after hyperparameter optimisation. FCNet is designed to avoid overfitting by including a dropout layer after each fully connected (Fc) layer.

FCNets are used as a generic framework for some inputs such as text and extracted features because of their robustness. Therefore, a 1D vector of the histogram-based features was fed to our four-layer FCNet architecture when reducing the layer sizes (Figure 11). Also, to avoid overfitting, our network was formed so that a dropout layer followed each fully connected layer. The *SoftMax* layer was connected to the last fully connected layer with *Fc* = 5 for the classification of the five different stages (cf. Figure 9). Two-hundred and forty filters were used for the first convolution and *Drop out* = 0.5 was chosen after optimization.

#### Convolutional neural networks

**Figure 12.**
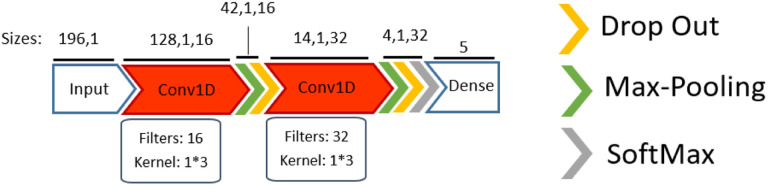
The proposed 1D-Conv architecture after hyperparameter optimisation. In 1D-Conv, max pooling is applied to reduce the dimensionality.

The input of our proposed 1D-Conv network with *kernel size* = 3, *max pooling* = 3, and *stride* = 3 is a 1D vector of the histogram-based features. To reduce the negative effect of overfitting, the output of the last dropout layer was flattened into a 1D vector, and the fully connected layer reduced the dimensionality using a max pooling before feeding into a *SoftMax* layer for classification. Figure 12 demonstrates the architecture of the proposed 1D-Conv. Sixteen and 32 filters were chosen for the first and second convolution, respectively, after optimization.

We created a long 1D vector: ${\text{hist}}_{{\text{combined}}_{-1D}}==[{\text{hist}}_{\text{part}1},{\text{hist}}_{\text{part}2},\ldots ,\{{\text{hist}}_{\text{partn}}]$ by appending histogram features of individual body parts to generate bilateral fused features for Hands, Knees, Feet, and Full-body. To maximise the use of spatial information between different body parts during movement, we proposed an additional 2D-CNN. In the latter, a 2D matrix shape called ${\text{hist}}_{{\text{combined}}_{-2D}}$ is formed by reshaping the 1D feature vector to a 2D matrix (2). Each row of the 2D matrix contains the histogram of features derived from a single body part.


(2) $${\text{hist}}_{{\text{combined}}_{-2D}}=\left[\begin{array}{c} {\text{hist}}_{\text{part}1}\\ {\text{hist}}_{\text{part}2}\\ \cdot \\ \cdot \\ \cdot \\ {\text{hist}}_{\text{partn}} \end{array}\right]$$

**Figure 13.**
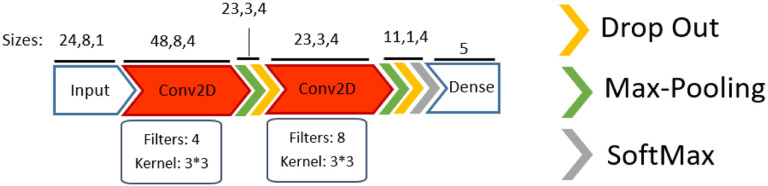
The proposed 2D-Conv architecture.

As shown in Figure 13, the proposed 2D-Conv consists of two 2D convolution layers followed by a max-pooling and dropout. In this architecture, kernel size was 3 and stride was 1 for convolutions, and the output was downsampled with max-pooling using the same kernel size and *stride* = 2. The output of the final dropout layer was flattened to a 1D vector, which was then reduced in dimension by a fully connected layer before being fed into a *SoftMax* layer for classification. Four and eight filters were chosen for first and second convolution, respectively, after optimization.

#### Capsule Neural Networks

To prevent losing information when using pooling in CNNs, we leveraged the CapsNet architecture, which utilises a novel technique called Dynamic Routing (DR) to establish an optimal path between different capsule layers (**Algorithm.1** in Supplementary Information). This enables the construction of hierarchical relationships between features while requiring fewer training samples. Also, CNNs ignore the positional correlation among local features in the spatial domain due to their focus on translation invariance and parameter sharing for efficient and robust representation learning.

**Figure 14.**
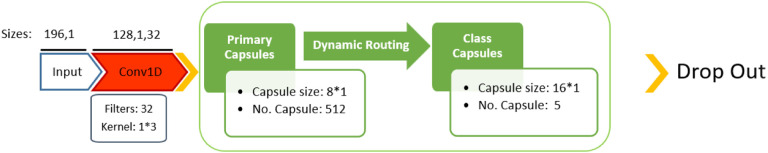
The proposed 1D-CapsNet architecture after hyperparameter optimisation.

**Figure 15.**
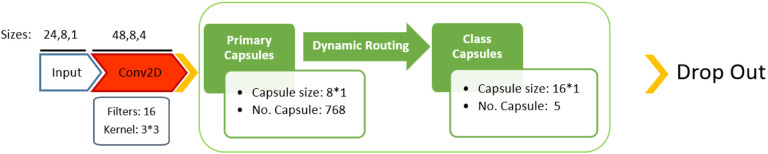
The proposed 2D-CapsNet architecture after hyperparameter optimisation.

To mitigate these issues, we used the same convolution layer settings for 1D and 2D CNNs, then removed the max-pooling before forming features and encapsulating them in Primary Capsules (PCs). Dynamic routing was then applied to send the most relevant primary capsule to the class capsule. Figures 14–15 display the proposed CapsNet architectures, which can receive both 1D and 2D histograms as input. As stated in results, we can either generate a 1D-CapsNet or a 2D-CapsNet, depending on the input dimension. After parameter optimization, *Kernel filter* = 3 was used for both CapsNet models, while 32 and 16 filters were chosen for the number of filters in 1D- and 2D-CapsNet, respectively, in convolution layers.

### Parameter Settings

We used five subjects with uniform joint landmarks from the experiment, and a leave-one-out (*i.e*., one subject) cross-validation approach. In addition, to better understand the temporal evolution of infant movement, an overlapped window sliding strategy was employed that used a window width of 500 frames with 100 overlapped frames across all experimental stages. This allowed us to assess fluctuations in classification accuracy across time throughout the experiment (illustrated with small black windows and arrows in Figure 9). Average classification accuracy on sliding windows is reported as a measure of model performance. Table 1 lists the hyperparameters optimised for each model. Since no hyperparameter optimisation was employed in previous work^55^, we used a range of parameters to generalise approaches (Table 1). For FCNets, we optimised the hyperparameters using FCNet layers = (240,60,15) and *dropout rates* = (0.5,0.7,0.9). The first layer filters (8,16,32) and the second layer filters (8,16,32) for the 1D-Conv approach were also optimised. Similarly, to 1D-Conv, we chose the following first- and second-layer filters for 2D-Conv = (4,8,2), *Kernel size* = (3,4,5), and the number of filters = (8,16) for optimising parameters in both the 1D and 2D-CapsNet approaches.

## Results

### Classification accuracies

Table 2 presents the average classification accuracy for the various machine and deep learning approaches tested. The approximate chance level for accurately classifying the five experimental stages used here is 20%. Table 2 also reports accuracy levels for four fused features. This set of fused features was created by concatenating the histogram features based on the number of joints available in the datasets, forming a long 1D vector. The four resultant fused joints were (1) **Hands:** fused features from L&R hand, (2) **Knees:** fused features from L&R knee, (3) **Feet:** fused features from L&R foot, and (4) **Full-body:** fused features from L&R hand, L&R knee, and L&R foot.

The classification accuracy achieved for L/R foot individually and fused Feet was superior to that of all other feature sets. In particular, 2D-CapsNet achieved the highest accuracy of 86.25% for the fused Feet feature set. In terms of mean joint type accuracy, all foot features (*i.e*., L, R and fused) scored significantly higher average accuracy across all evaluated classification methods compared to other joint types (*p* < 0.0005).

LDA and kNN achieved highest accuracy for single joint features. For example, LDA achieved the highest accuracy of 59.63% and 75.63% for the left hand and left foot, respectively, whereas kNN achieved 58.00% and 61.63% for the right hand and left knee, respectively.

However, when we considered fused features, deep learning approaches outperformed LDA and kNN. All fused features improved in accuracy using deep techniques. When spatial information between different body parts was used during movement, 2D deep approaches performed better: 2D-Conv and 2D-Capsnet achieved an accuracy of 59.57% and 86.25% for Hands and Feet, respectively. Even though 2D-CapsNet achieved a lower accuracy for Hands (50.65%), a comparison of the mean accuracy per classifier demonstrated that 2D-CapsNet maintained the highest mean accuracy at 65.65. Also, the fused feature of Full-body that represent the most general representation of 3D skeletal joint information achieved the highest accuracy (65.51%) using the 2D-CapsNet approach. In sum, though all models tested performed well above chance (20%), 2D-CapsNet models using fused feature inputs made the most accurate classifications.

### Using the 2D-CapsNet to assess stage transitions

**Figure 16.**
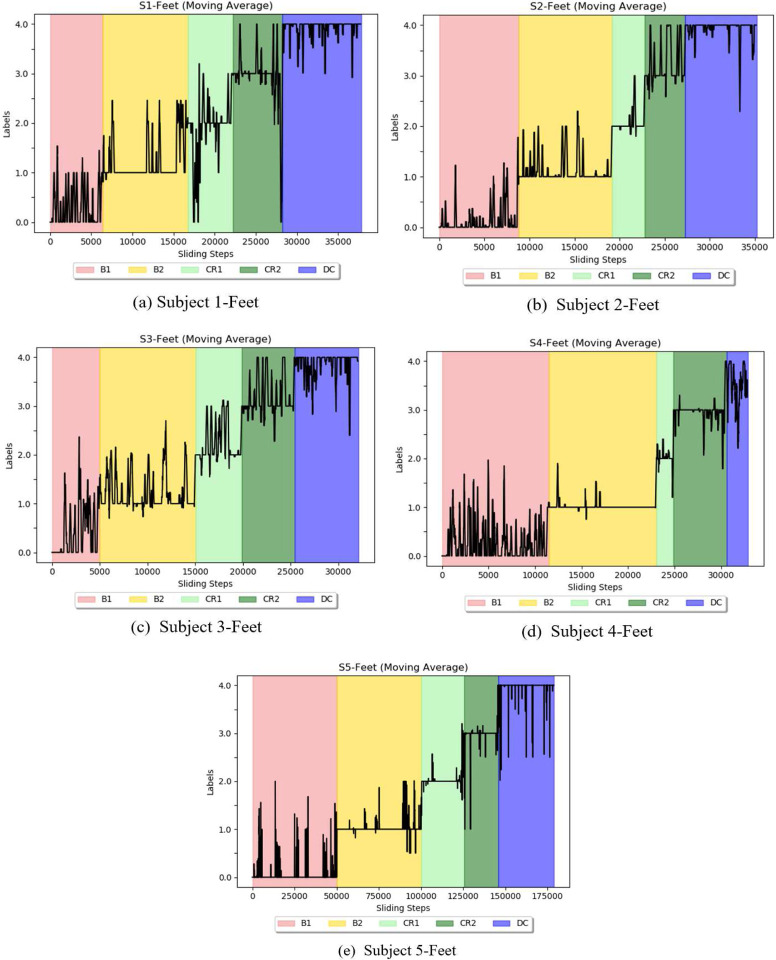
Temporal analysis for each subject. Five ascending steps illustrate the correct labels for five distinct classes. Labels 0, 1, 2, 3, and 4 on the Y axis correspond to stages B1, B2, CR1, CR2, and DC, respectively.

**Figure 17.**
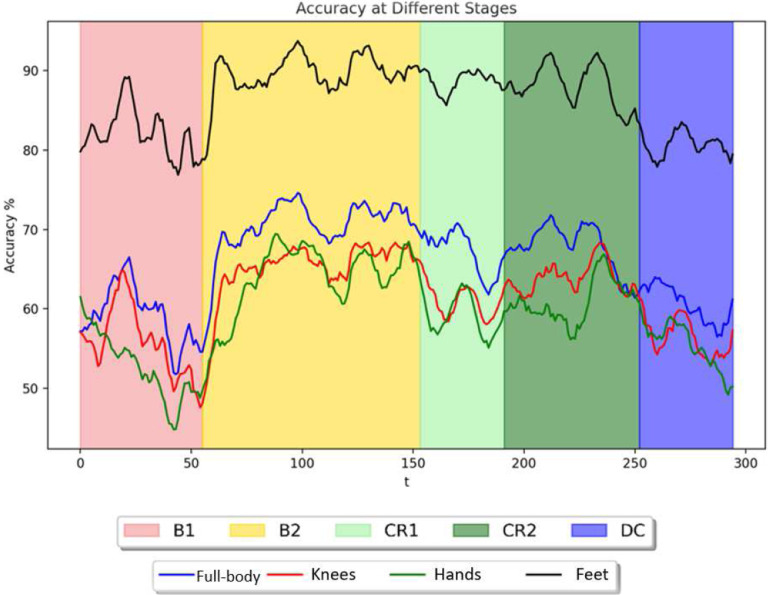
The moving average of the classification accuracy for temporal analysis of fused features (Full-body, Knees, Hands, and Feet).

A sliding window was used to analyze each infant’s movement in conjunction with the leave-one-out approach. For each infant, we shifted a window of 500 frames with 100 frames overlapping across one stage while keeping other windows stationary in the remaining stages. Figure 16 (a–e) shows the moving average of the temporal analysis for each infant using the fused Feet feature. As can be observed, five ascending steps illustrate the correct labels for five distinct classes. Labels 0,1,2,3, and 4 on the Y axis correspond to stages B1 (spontaneous baseline, no mobile motion), B2 (experimenter triggered mobile motion), CR1 (minute 1 of coupling), CR2 (minute 2 of coupling), and DC (decoupled, no mobile motion), respectively. Stage B2 received the most accurate labels, indicating that it is easier to detect Histograms of Joint Displacements (HJDs) accurately compared to other stages. Additionally, the classifier did not perform well when it came to identifying spontaneous movement in stage B1, indicating that infants moved variably such that the system detected movements in B1 which were like movement patterns in multiple other stages. This becomes more evident when considering the moving average of classification accuracy for temporal analysis of fused features Knee, Hand, and Feet (Figure 17). B2 has a higher overall classification accuracy over the whole period than the preceding stage. Additionally, as we moved along after decoupling (in stage DC), we observed a decline in classification accuracy, indicating that movements were more irregular after the mobile stopped responding to infant movement.

### Feature analysis

We first computed the average displacement rates of both feet across the stages to further understand the significance of higher classification accuracy levels observed during B2 and across CR (see previous section). As can be seen in Table 3, the feet were significantly more active during DC compared to the other stages (*F*(4, 16) = 3.52, *p* = 0.03, sphericity assumed), and infants tended to move their feet less during B2 compared to the other stages. Based on average foot movement rate alone, accurately classifying B2 and DC foot behaviour would appear to be easier task compared to classifying stages B1 and CR which had similar movement rates.

**Figure 18.**
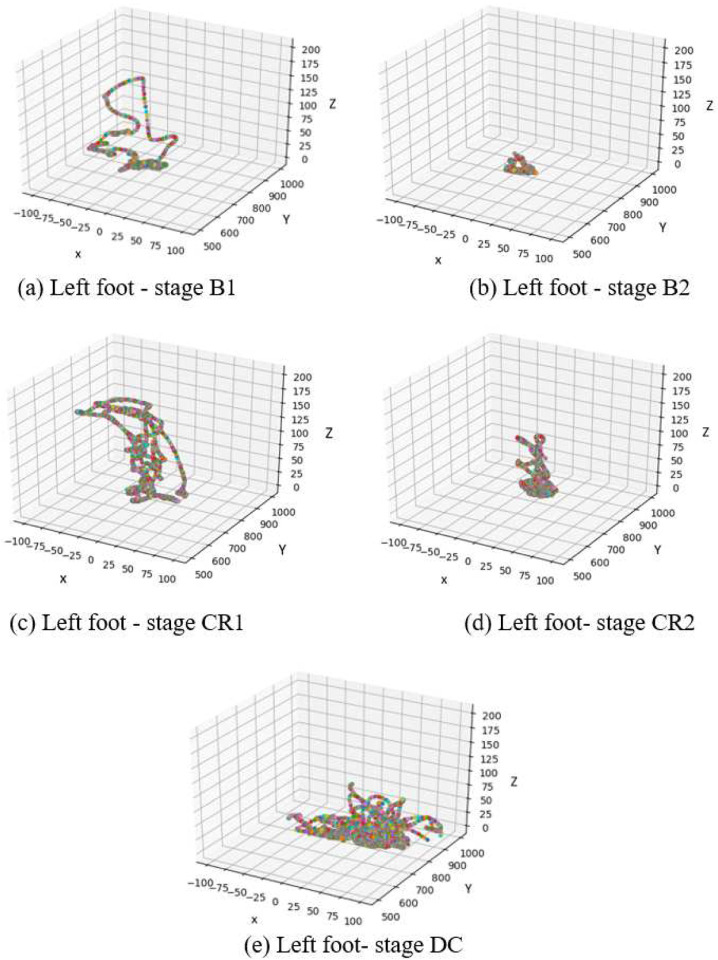
The trajectory of the left (connected) foot in S1 as a function of experimental stage.

Infant learning during coupling has classically been defined as 150% increase in movement rate relative to spontaneous baseline^8^. While this cut-off was not met by these five babies as a group, the infants do increase movement rate roughly 150% during tethering relative to the second baseline when the experimenter moved the mobile. Given that both the coupled phase and second baseline both involve mobile movement, the second baseline is a fair comparison point contextually speaking. Furthermore, averaging movement rate across babies does not address individual behaviour or discovery processes. Finally, it is possible that infants discover specific trajectories that are particularly suited to elicit mobile response. Upon discovering the relationship between foot movement and mobile motion, infants may adjust foot movement topology rather than (or in addition to) foot movement rate. These infants do in fact change their foot trajectories as is reflected by the high accuracy rates depicted in Figure 18

**Figure 19.**
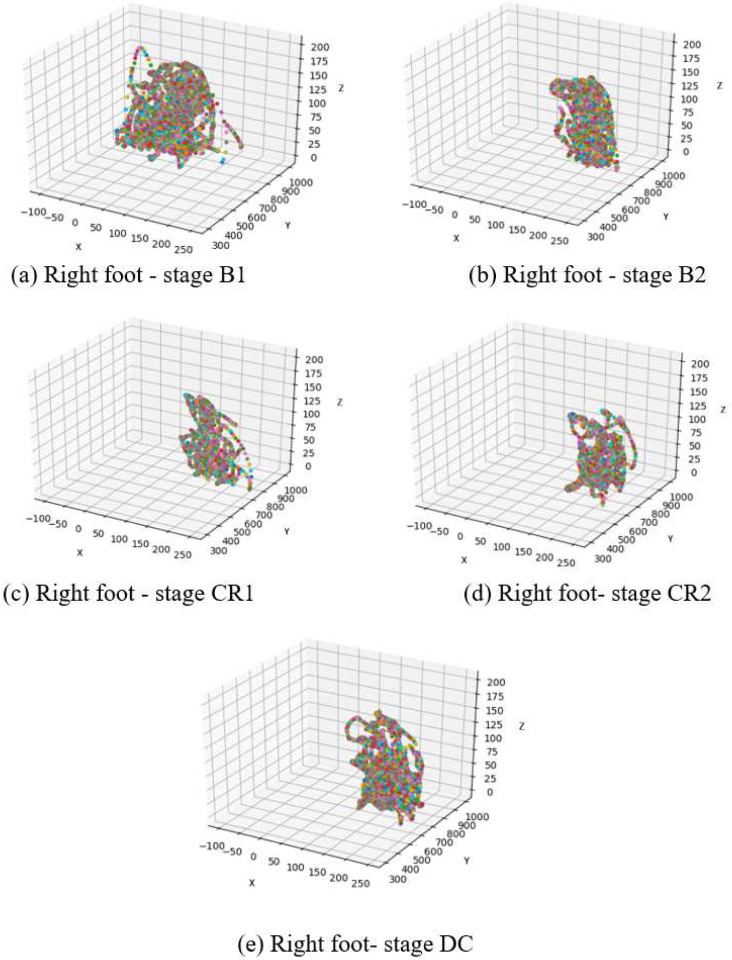
The trajectory of the right (connected) foot in S2 as a function of experimental stage.

To explore topological changes in infant joint movement, we examined the actual trajectory of the connected feet for Subject 1 (left foot) and Subject 2 (right foot) (Figures 18–19). As can be observed, random movements in stage B1 became more constrained in both subjects after the examiner activated the mobile (B2). Topologically, infants produced almost the same movement trajectories for stages CR1 and CR2. S2 maintained similar activity in DC compared to CR, indicating lasting effects of baby-to-world connection even after that connection is severed.

**Figure 20.**
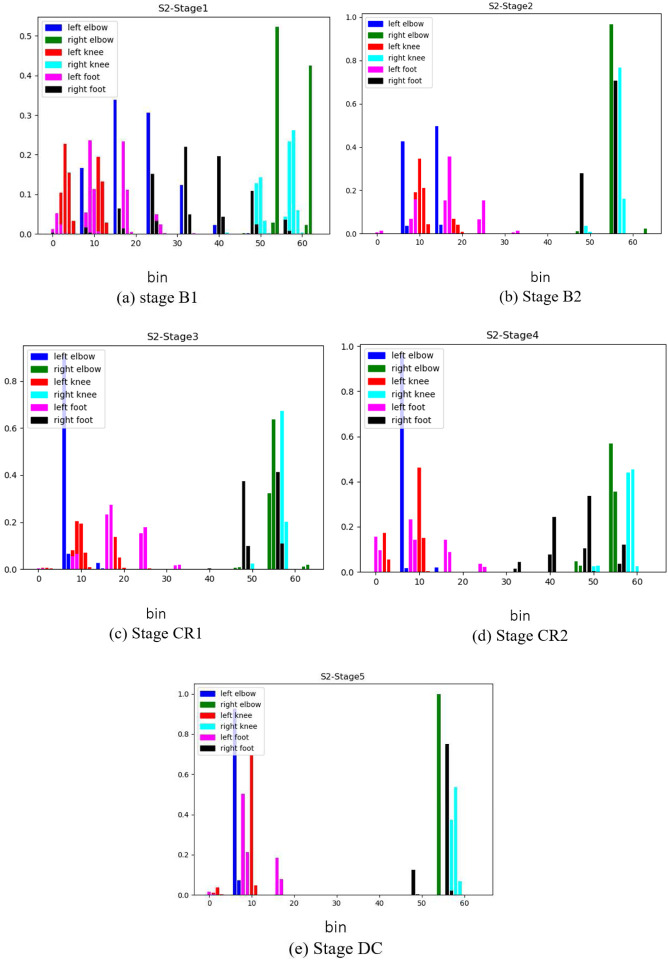
The HJD for Full-body joints over 1000 frames in the middle of each stage for S2. The x-axis is the number of the 64 bins used in the modified spherical coordinate system.

To investigate how changes to features of infant movement directly inform deep learning systems, we quantified the HJDs of all joints for S2 over 1000 frames in the middle of each stage (*bins* = 64) (see Figure 20). Note that whereas the x-axis of each histogram reflects the number of bins (or the range of 3D space) a joint occupies, the y-axis reflects the number of frames the joint occupied a particular bin. Therefore, a large HJD value constrained to a single bin reflects little joint motion or, at most, very topologically restricted motion. Although most joints during B1 are clearly separated to the left and right of the histogram graph space in accordance with their anatomical sides (*e.g*., left hand (blue) occupies bins on the left of the histogram), the right foot (black) is visible in many bins between bins 8−57, implying random movement of the right foot in stage B1. The right foot movement became more restricted throughout stage B2 (bins 48 and 56), supporting the observations made in the preceding section (Results, C). There was a noticeable increase in the range of this joint’s position across the CR stage (activity was between bins 46−57 in CR1 and expanded between bins 32−57 in CR2). Although the foot was active in a larger volume in CR than B2, activity became constrained again in the DC stage, like B2. Importantly, this evolution of activity was only observed in the foot linked to the mobile (the right foot) and not in any other limb segments.

## Discussion

### Comparison of AI approaches: Which AI approach works best and why?

We aimed to use machine and deep learning approaches to identify infant behavioural changes in response to changes in functional context. Both machine and deep learning techniques accurately classified infant Pose-Based features (HJDs) throughout the experiment. All techniques tested reached accuracies higher than chance (20%) for all joint types (Table 2), although deep techniques generally outperformed machine learning techniques for any given joint type (Table 2). In particular, 2D-CapsNet achieved the greatest accuracy level (accuracy for fused Feet= 86%). Thus, 2D-CapsNet maximised spatial information between different body parts during movement, demonstrating the architecture’s high-performance ability to generate feature hierarchy.

### Comparison of joint classification across AI approaches: Which joint has the most distinctive patterns and why?

For every architecture tested, whether of machine or deep learning, some measure of the feet achieved the highest classification accuracy rates (∼20% higher than the fused hands, knees or whole body), indicating that the feet had the most distinctive pattern changes across the various experimental stages in response to the mobile’s changing behaviour and the infant’s functional connection to the mobile.

### Do dynamics of classification accuracy reflect infant discovery?

The classification accuracies of several joints were assessed continuously over time to investigate how infants adapt to functional context and whether accuracy dynamics indicate that infants transition from exploring their relationship with the mobile to intentionally directing mobile movement. Again, classification accuracy was strongest by far for the feet, the end effector, compared to other body parts regardless of experimental stage (Figure 17). Accuracy of the feet fluctuated around 80% during the spontaneous baseline, a lower level than when the mobile moved either at the hand of the experimenter during the second baseline or at the foot of the infant during coupling. These results taken together with the large spread seen in the first baseline histogram of one infant (each of Subject 2’s feet appear in 10 histogram bins, Figure 20a) denote that infants’ movements are more widespread topologically and therefore harder to classify when the infant is moving spontaneously without any active environmental stimulation. On the other hand, once the experimenter began triggering the mobile, classification accuracy jumped up, hovering around 90% throughout the second baseline. This spike in classification accuracy is related to the concentration of foot activity within 3D space during the second baseline (*e.g*., each of Subject 2’s feet appear in only one or two histogram bins, Figure 20b). Soon after infant∼mobile coupling began, there was a momentary dip in accuracy (Figure 17), possibly reflecting infants’ initial probing of a variety of trajectory orientations. However, accuracy levels spiked in the second minute of coupling (hovering again around 90%), likely reflecting infant discovery of trajectories particularly suited for triggering mobile motion (*e.g*., the switch to Z-orientation seen in individual infants as they raised and lowered their foot to trigger mobile motion, Figures 18–19). In sum, classification accuracy fluctuates within and across experimental stages and seems to reflect processes of exploration and discovery. However, what can we make of the fact that classification accuracy levels were similar when the experimenter triggered the mobile and during coupled interaction?

Since histograms of joint displacement were used as inputs to the AI architectures tested here, stark changes in overall quantity of limb activity from stage to stage are likely to be relevant to stage classifications. Accurately classifying foot behaviour based on average foot movement rate alone seems to be more straightforward during the second baseline (when infants were least active compared to the spontaneous baseline and the coupled stage as the latter two stages had similar movement rates (Table 3)). Therefore, although similar classification accuracy rates were obtained for the feet in the second baseline and during coupling, the high classification accuracy results during the second baseline are largely explained by the overall reduction in movement rate. In contrast, the high classification accuracy during coupling indicates that foot movement changes topologically during coupling and across the stages. Our AI architectures use those qualitative changes (not merely quantity of movement) to classify behaviour. Though infant contingency studies traditionally infer learning when movement quantity is much greater during coupling compared to spontaneous baseline, our results also demonstrate that qualitative changes in infant movement (Figures 16–20) can be detected even when average movement rates do not differ significantly (Table 3).

After the connection between infant and mobile was severed, activity spiked (Table 3) and accuracy quickly declined, plunging even lower than spontaneous baseline (Figure 17). Infants appear to switch from directed movement during coupling to varied, exploratory movement after decoupling. As a group, they explore to a greater degree after being disconnected than before they were exposed to the possibility of controlling the mobile. It is as if the infants’ search for connection to the world has intensified because of the lost linkage. However, certain infants’ trajectories in the decoupled stage reflect a lasting imprint of coupling (*e.g*., S2, Figure 19). It is possible that only some infants recognize their functional relationship to the mobile and so only these infants maintain the patterns which emerged during coupling after decoupling, expecting those unique patterns to elicit the mobile response. If the accuracy rate during decoupling persists at the same high level observed during coupling or dips below the lower accuracy rate seen during spontaneous movement, either pattern could indicate infant discovery during coupling. Using classification accuracy to identify infants who discover their ability to make the mobile move is complicated by the fact that different behaviours could indicate infant discovery for different reasons. Identifying infant discovery likely requires piecing together clues from various experimental stages. Therefore, temporal analysis of classification accuracy for each infant across the entire experiment (Figure 16) provides an important tool for understanding individualized processes of exploration and discovery.

### Limitations and future directions

As in any formal comparison of machine and deep learning techniques, future research may explore the effects of other hyperparameter settings on classification accuracy rates. Although the histogram-based techniques used here condensed 3D movement data to a lower dimensionality by grouping data into bins, a comprehensive representation of the data was still preserved. Also, movement was represented using feature descriptors that are intended to simplify and extract useful information. As a result, rather than performing a frame-by-frame comparison, we were able to examine the distribution of the displacement of all joints across a time window. Future studies may incorporate the actual recorded video and skeletal joint information involving more infants and employ Recurrent Neural Networks (RNN) and more advanced Transformer and attention-based models to investigate the feasibility of predicting or decoding the temporal information of the input posture sequence. In addition, other advanced pose-based features can be used to evaluate the proposed network architectures^58^.

## CONCLUSION

Both machine and deep learning techniques successfully classified randomly sampled five-second snippets of 3D infant movement from different experimental stages using histogram features of pose-based information. The deep learning 2D-CapsNet, however, achieved the highest accuracy classification rate. Critically, for every architecture tested, whether of machine or deep learning, some measure of the feet achieved the highest classification accuracy rates. Without informing these AI systems about the experimental design or the connectivity of the foot to the mobile, every architecture tested speaks the same message: the feet, the end effectors, are most uniquely affected by the baby∼mobile interaction. Put another way, the functional connection to the world influences the baby most where it matters, namely at the point of infant∼world connection. The information flow between agent and world is palpable; it is bodily felt and enacted. In contrast to traditional infant contingency studies which rely upon changes in movement quantity to infer learning, our AI classifiers offer a means to automatically detect characteristic topological changes across the experimental stages. Thus, AI systems can play a significant theoretical role, namely by providing insight into the early ability of infants to actively detect and engage in a functional relationship with the environment. Additionally, assessing dynamics of AI classification accuracy for each infant opens a new avenue for unravelling when and how individuals engage with and discover their relationship to the world. Whereas previous AI approaches have focused on classifying spontaneous infant movement in relation to clinical outcomes, pairing theory-driven experimentation with AI tools will ultimately allow us to develop more robust, context-dependent and functionally relevant assessments of infant behaviour for risk, diagnosis and treatment of disorders.

## Figures and Tables

**Table 1.** Hyper-parameters selection.

Models	Hyper-parameter 1	Hyper-parameter 2
**FCNet**	# first FCNet layer: (240, 150, 100)	Dropout rates: (0.5, 0.7, 0.9)
**1D-Conv**	# first-layer filters: (8, 16, 32)	# second-layer filters: (8, 16, 32)
**2D-Conv**	# first-layer filters: (4, 8, 32)	#second-layer filters: (4, 8, 32)
**lD-CapsNet**	Kernel size: (3, 4, 5)	# layer filters: (16, 32)
**2D-CapsNet**	Kernel size:(3,4, 5)	# layer filters: (8, 16)

**Table 2.** Performance of all models: average sliding window accuracy (%).

Joint-Type	Classification Accuracy							
	LDA	Knn	FCNet	1D-Conv	1D-CapsNet	2D-Conv	2D-CapsNet	MEAN Joint-Type accuracy
**Left hand**	**59.63%**	55.89%	50.15%	55.57%	55.12%	-	-	55.27%
**Right hand**	51.15%	**58.00%**	51.26%	57.84%	50.32%	-	-	53.72%
**Hands**	52.63%	54.89%	55.25%	59.19%	56.55%	**59.57%**	50.65%	55.53%
**Left foot**	**75.63%**	64.84%	71.63%	70.10%	60.89%	-	-	68.61%
**Right foot**	71.31%	62.68%	**77.78%**	61.21%	68.24%	-	-	68.24%
**Feet**	70.63%	63.34%	73.62%	78.15%	81.15%	65.65%	**86.25%**	**74.11%**
**Left knee**	39.05%	**61.63%**	53.05%	58.78%	58.25%	-	-	54.15%
**Right knee**	50.10%	**59.42%**	51.55%	59.26%	57.14%	-	-	55.49%
**Knees**	50.55%	33.60%	51.23%	59.78%	**61.22%**	59.66%	60.19%	53.75%
**Full-body**	39.63%	50.89%	57.88%	56.52%	60.60%	56.12%	**65.51%**	55.31%
**MEAN Classifier Accuracy**	56.03%	56.52%	59.34%	61.64%	60.95%	60.25%	65.65%	-

* For each joint-type, the model with greatest classification accuracy is in bold.

** For each model, the joint-type with greatest classification accuracy is in red.

**Table 3.** Average Displacement Rate of Feet (m/min).

Stage					
	B1	B2	CR1	CR2	DC
Mean	15.54	9.95	14.46	14.70	18.65
*SE*	5.55	4.48	3.13	3.80	3.85

## Data Availability

The datasets used and/or analysed during the current study are available from the corresponding author on reasonable request.
